# Vulnerabilities at First Sex and Their Association With Lifetime Gender-Based Violence and HIV Prevalence Among Adolescent Girls and Young Women Engaged in Sex Work, Transactional Sex, and Casual Sex in Kenya

**DOI:** 10.1097/QAI.0000000000001826

**Published:** 2018-08-10

**Authors:** Marissa L. Becker, Parinita Bhattacharjee, James F. Blanchard, Eve Cheuk, Shajy Isac, Helgar K. Musyoki, Peter Gichangi, Sevgi Aral, Michael Pickles, Paul Sandstrom, Huiting Ma, Sharmistha Mishra

**Affiliations:** * Center for Global Public Health, Department of Community Health Sciences, University of Manitoba, Winnipeg, MB, Canada;; † Technical Support Unit, Partnership for Health and Development in Africa, Nairobi, Kenya;; ‡ India Health Action Trust, Bangalore, India;; § National AIDS and STI Control Programme, Nairobi, Kenya;; ║ International Center for Reproductive Health Kenya, Mombasa, Kenya;; ¶ Centers for Disease Control and Prevention, Atlanta, GA;; # Retrovirology Laboratory, JC Wilt National HIV, Winnipeg, WB, Canada;; ** Li Ka Shing Knowledge Institute, St. Michael's Hospital, University of Toronto, Toronto, ON, Canada; and; †† Division of Infectious Disease, Department of Medicine, University of Toronto, Toronto, ON, Canada.

**Keywords:** adolescent girls and young women, female sex workers, transactional sex, HIV, violence, Kenya

## Abstract

**Background:**

Adolescent girls and young women (AGYW) experience high rates of HIV early in their sexual life course. We estimated the prevalence of HIV-associated vulnerabilities at first sex, and their association with lifetime gender-based violence (GBV) and HIV.

**Methods:**

We conducted a cross-sectional biobehavioral survey among AGYW (14–24 years) in Mombasa, Kenya in 2015. We compared the prevalence of first sex vulnerabilities across AGYW who self-identified as engaging in sex work (N = 408), transactional sex (N = 177), or casual sex (N = 714) and used logistic regression to identify age-adjusted associations between first sex vulnerabilities and outcomes (GBV after first sex; HIV).

**Results:**

The median age at first sex was 16 years (interquartile range 14–18). A total of 43.6% received gifts or money at first sex; 41.2% and 11.2% experienced a coerced and forced first sex, respectively. First sex vulnerabilities were generally more common among AGYW in sex work. GBV (prevalence 23.8%) and HIV (prevalence 5.6%) were associated with first sex before age 15 [GBV adjusted odds ratio (AOR) 1.4, 95% confidence interval (CI): 1.0 to 1.9; HIV AOR 1.9, 95% CI: 1.1 to 1.3]; before or within 1 year of menarche (GBV AOR 1.3, 95% CI: 1.0 to 1.7; HIV AOR 2.1, 95% CI: 1.3 to 3.6); and receipt of money (GBV AOR 1.9, 95% CI: 1.4 to 2.5; HIV AOR 2.0, 95% CI: 1.2 to 3.4).

**Conclusions:**

HIV-associated vulnerabilities begin at first sex and potentially mediate an AGYW's trajectory of risk. HIV prevention programs should include structural interventions that reach AGYW early, and screening for a history of first sex vulnerabilities could help identify AGYW at risk of ongoing GBV and HIV.

## BACKGROUND

Adolescent girls and young women (AGYW) experience a disproportionate burden of HIV-associated vulnerabilities leading to a disproportionate risk of HIV acquisition.^1^ This is particularly prominent in regions with high HIV prevalence such as Eastern and Southern Africa, where women aged 15–24 years comprise 10% of the adult population yet accounted for 26% of new HIV infections in 2016.^2^ In Kenya, overall HIV prevalence is 5.4% and AGWY accounted for 32% of the estimated 56,000 new adult HIV infections in 2016.^3^

A complex interplay of biological, behavioral, and structural factors contributes to the increased vulnerability to HIV acquisition among AGYW. The timing of these HIV-associated vulnerabilities and a woman's first sexual experience may influence her HIV risk.^4^ Early age at first sex (often defined as <15 years of age^5^) is associated with increased risk of bacterial sexually transmitted infections partly due to cervical ectopy in adolescence,^6,7^ higher levels of inflammatory cytokines in the female genital tract,^8,9^ less condom use at first sex and subsequent sex acts,^10,11^ and prevalent HIV infection.^4,12^ AGYW who reach menarche and puberty early are more likely to engage in earlier (and often condomless) sexual activity than those who experience later menarche.^13–15^ Sex with older men is common among AGYW, and is associated with increased HIV risk because older men are more likely to be living with HIV than younger men, and because the age-disparate and gender-based power dynamics undermine condom negotiation.^16–19^ Structural factors including intimate partner violence and other forms of gender-based violence (GBV) are associated with HIV acquisition,^20–22^ and GBV is common among AGYW.^23,24^

AGYW engage in a spectrum of sexual partnerships—paid, transactional, casual, and intimate—across which early HIV-associated vulnerabilities and HIV prevalence may vary. In sub-Saharan Africa, women engaged in sex work (SW) experience a 12-fold higher prevalence of HIV compared with other women of reproductive age^25,26^, and transactional sex (TS) outside of formal SW among women aged 13–26 years is associated with a 2-fold higher risk of HIV compared with those who never engaged in TS.^27^ As with AGYW in general, evidence suggests that HIV acquisition in the context of SW occurs early—either before or during the first few years after a person self-identifies as a sex worker.^28^ Earlier age of entry into SW (before versus after age 17) is associated with inconsistent condom use, experiences of violence, and higher rates of HIV, but less is known about the circumstances surrounding first sex among young female sex workers.^29–32^

Limited data exist on the prevalence of HIV-associated vulnerabilities at first sex across AGWY engaged in various types of sexual partnerships, and how first sex vulnerabilities are associated with ongoing vulnerabilities and with HIV prevalence.^4^ Most data on the circumstances surrounding first sex in sub-Saharan Africa come from household surveys, which may not capture AGYW engaged in SW, and are limited to age at first sex and menarche.^4,5,14,33^

We sought to estimate the prevalence and distribution of first sex vulnerabilities and their association with lifetime experience of GBV and with HIV prevalence, across subgroups of AGYW in Mombasa, Kenya, engaged in SW, TS, and casual sex (CS).

## METHODS

We conducted a cross-sectional biological and behavioral survey between April and November 2015, among sexually active young women age 14–24 years old in Mombasa, Kenya.

### Study Setting and Population

Before survey implementation, we used geographic mapping^34,35^ to estimate the number and distribution of AGYW aged 14–24 years congregating at SW “hotspots”—defined as locations where female sex workers solicit clients such as bars, night clubs, hotels, and public spaces. The mapping generated the sampling frame, and we used a multistage cluster sampling approach with probability proportional to the size of the enumerated AGYW population in the hotspots.

### Sample Frame and Study Procedures

Community mobilizers (former or current female sex workers working with the International Center for Reproductive Health Kenya) identified potential participants at sampled hotspots and arranged for visits to the study sites where screening and enrollment occurred. Women were eligible to participate if they were between 14 and 24 years of age, sexually active (ever had vaginal or anal intercourse), and could provide written informed consent to trained interviewers at the study sites. Interviewers conducted face-to-face interviews with a structured questionnaire in English or Kiswahili.

A trained HIV testing counselor conducted the rapid HIV testing using fingerprick whole blood sampling as per the national HIV testing guidelines, with results immediately provided to the participant and referral for HIV treatment and care if the result was HIV-positive.

Participants could provide a venous blood sample as part of a nested biological substudy, from which a dried blood spot (DBS) specimen was made in the International Center for Reproductive Health Kenya laboratory at the Coast Provincial General Hospital in Mombasa or provide a DBS specimen at the study site from a fingerprick sample. DBS samples were transferred to the National HIV and Retrovirology Laboratories in Winnipeg, Canada, and underwent serological testing through the Avioq HIV-1 Microelisa System (Avioq Inc., Research Triangle Park, NC).

Questionnaire data were entered in to a CSPro database and linked to the DBS serology results.

### Definitions

The questionnaire included items about sexual partnerships, the answers to which we used to classify participants into 1 of 3 mutually exclusive groups. Participants were classified as engaging in SW if they self-identified as a sex worker, or ever reported sex in exchange for money wherein the price was negotiated before the sex event. Participants were classified as engaging in TS if they could not be classified as engaging in SW but reported at least 1 sex partner in their lifetime where there was an expectation and receipt of money/goods in return for sex but the price was not prenegotiated.^36^ The remainder of the participants were classified as engaging in CS. We defined hotspots as venue-based (bars, night clubs, hotels, guest houses, lodges, restaurants, local brew dens, sex dens, and brothels) and nonvenue-based (streets and other public places).

We conceptualized vulnerabilities at first sex based on their known association with HIV risk^16,17,20,24,29,37^ within the following domains: biological [age at first sex (vaginal or anal intercourse) less than 15; first sex before or within 1 year of menarche (calculated using age at first sex and age at menarche)]; behavioral (anal sex at first sex and condomless sex at first sex); partnership characteristics (first sex partner 10 years older, receipt of gifts/money for first sex, first sex arranged by someone other than respondent); and structural GBV (defined as a coerced or forced first sex event). Age at first sex was obtained from current age and duration of sexual activity (yrs/mo since first sex).

### Statistical Analysis

We reported descriptive statistics, including the prevalence and 95% confidence intervals (CIs) of vulnerabilities at first sex, and stratified by subgroups engaged in SW, TS, and CS. We compared proportions using χ^2^ and Fisher's exact tests as appropriate. We performed univariate and multivariate logistic regression, adjusting for age (as a continuous variable) to estimate the association between each first sex vulnerability and (1) lifetime experience of GBV (defined as sexual or physical violence experienced after first sex) and (2) HIV prevalence based on the DBS HIV serology results. We reported the crude odds ratio (COR) and adjusted odds ratio (AOR) accordingly. We restricted measures of association to cells with ≥5 participants.

We applied multiple imputation under assumption of missingness at random, to address missing data on age at first sex (N = 77 missing), and age of menarche (N = 30 missing). Covariates in the imputation model included current age, subgroup; literacy (can read and write); ever married; hotspot type; and each first sex vulnerability from the behavioral, partnership, and structural (GBV) domains. We generated 100 imputed data sets, the results of which were consistent with complete case analyses. We restricted analyses of the HIV prevalence outcome to the subset of participants who consented to HIV testing. We used R version 3.4.2 for imputation (mice package^38^), statistical analyses, and graphics.

### Ethics

The study was approved by the institutional ethics review boards of the University of Manitoba, Canada, the University of Nairobi, Kenya, and the National Commission for Science, Technology and Innovation, Kenya.

## RESULTS

### Participant Characteristics

Of the 1419 women screened, 1304 were eligible and invited to participate of whom 1299 (91.5% of those screened, and 99.6% of those eligible) consented and completed interviews (Table 1).

**TABLE 1.**
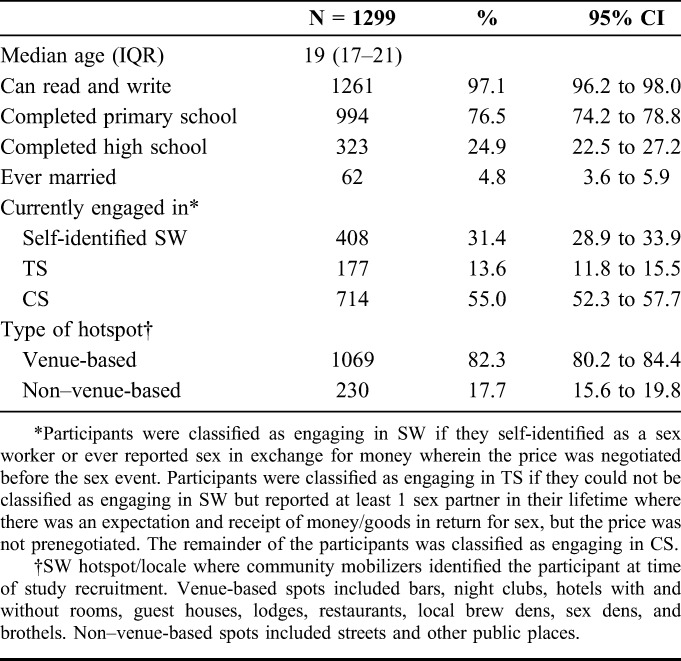
Characteristics of Study Participants Aged 14–24 Years in Mombasa, Kenya

Table 1 describes participant characteristics. There were 408 (31.4%), 177 (13.6%), and 714 (55.0%) participants in the SW, TS, and CS groups, respectively. Median age was 19 years [interquartile range (IQR) 17–21], and 82.3% of participants were recruited from venue-based hotspots. Most participants had completed primary school (76.5%), and few (4.8%) had ever married.

### Prevalence of HIV-Associated Vulnerabilities at First Sex

The median age at first sex was 16 years (IQR 14–18), and the age at first sex was <15 years among 27.8% of participants (Table 2). The median age of menarche was 14 years (IQR 13–15), and 39.0% of participants reported that first sex occurred before or within 1 year of menarche. All participants reported vaginal sex at first sex; anal sex at first sex was rare (0.8%). The majority (60.4%) of first sex acts were condomless. Age-disparate first sex partnerships were reported by 7.0% of participants, who recalled that their first sex partner was more than 10 years older than she was. Nearly half of participants received a gift or money at their first sex event (43.6%), of whom 54.1% received money. Many women (41.2%) felt coerced into their first sex and 11.2% reported a forced first sex.

**TABLE 2.**
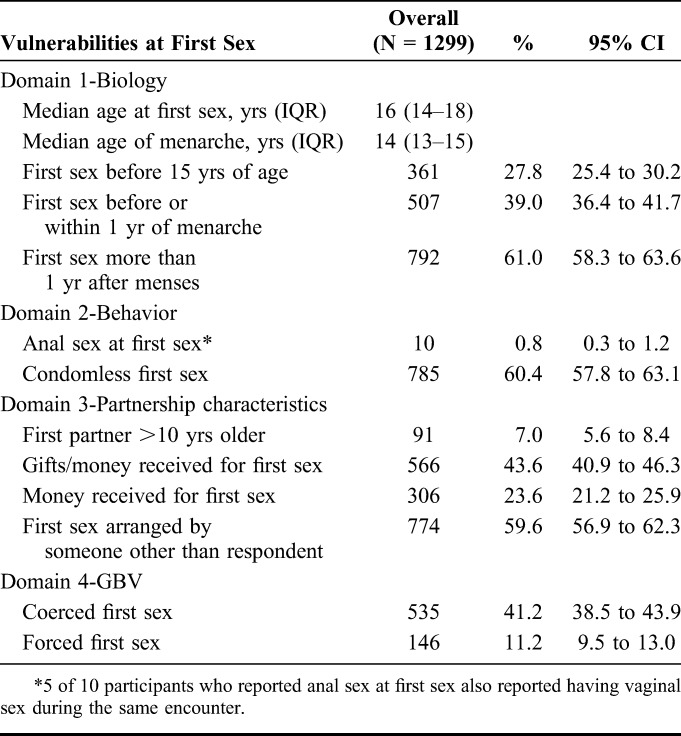
Prevalence of HIV-Associated Vulnerabilities at First Sex Among Women Aged 14–24 Years in Mombasa, Kenya

### Prevalence of First Sex Vulnerabilities by Current Engagement in SW, TS, and CS

**FIGURE 1.**
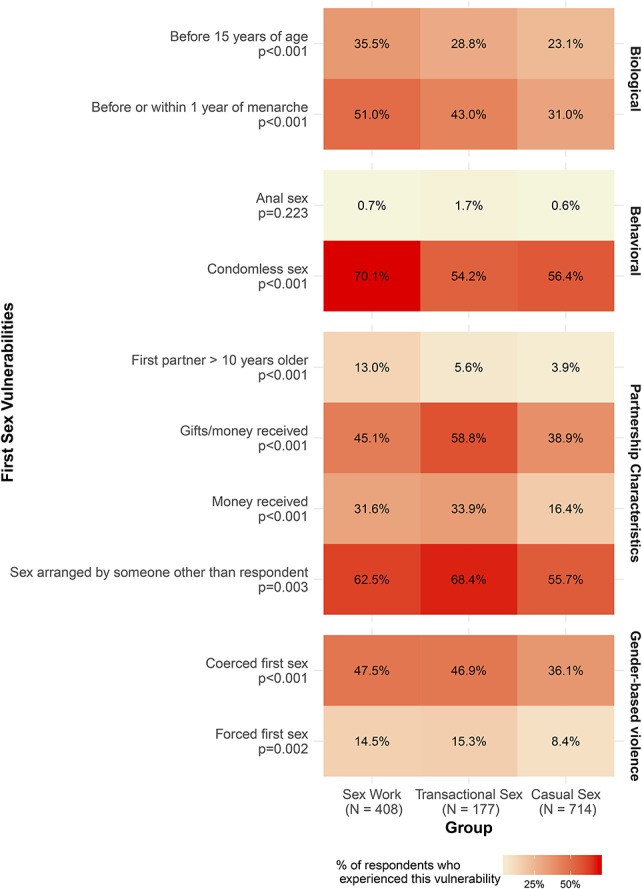
Distribution of vulnerabilities at first sex by current sexual partnerships among women aged 14–24 years in Mombasa, Kenya.

The prevalence of the first sex vulnerabilities varied significantly between subgroups (*P* < 0.005, Fig. 1) across all domains, with the exception of anal sex at first sex.

In the biological domain, first sex before age 15 was most commonly reported by SW (35.5%), followed by TS (28.8%), and then CS (23.1%, *P* < 0.001). The pattern was similar for a first sex before or within a year of menarche (Fig. 1) with the highest prevalence seen among SW (51.0%), followed by TS (43.0%) and then CS (31.0%).

In the behavioral domain, 70.1% of participants engaged in SW compared with just over half of participants engaged in TS or CS reported a condomless first sex (*P* < 0.001).

The partnership domain revealed different patterns between subgroups, with the highest prevalence of some vulnerabilities among participants engaged in TS. Older first sex partners were more common among participants engaged in SW (13.0%), followed by TS and CS (*P* < 0.001). However, participants currently engaged in TS were more likely than those engaged in SW to report an exchange of gifts or money at first sex (58.8% versus 45.1%, *P* < 0.001), followed by 38.9% in CS (between-group comparison, *P* < 0.001). Participants engaged in TS (68.4%), followed by SW (62.5%) and CS (55.7%), were most likely to report a first sex event arranged by someone else (*P* < 0.005).

In the GBV domain, nearly half of participants engaged in SW (47.5%) and TS (46.9%), respectively, reported a coerced first sex (*P* < 0.001), followed by 36.1% in CS (between-group comparison, *P* < 0.001). Reports of a forced first sex were also similar between participants engaged in SW (14.5%) and TS (15.3%, *P* = 0.940), and lowest among CS (8.4%) (between-group comparison, *P* < 0.005).

### Association Between First Sex Vulnerabilities and Lifetime GBV and HIV Prevalence

Tables 3 and 4 show the associations between first sex vulnerabilities and lifetime experience of GBV and HIV prevalence. One in 4 participants experienced GBV after first sex (23.8%, 95% CI: 21.5% to 26.1%), with a prevalence of 37.5%, 32.2%, and 13.9% among those engaged in SW, TS, and CS, respectively (*P* < 0.001, data not shown). Among the 1193 participants who consented to DBS HIV testing, HIV prevalence was 5.6% (95% CI: 4.3% to 7.0%) and highest among those engaged in SW (10.1%), followed by TS (3.6%), and CS (3.6%, *P* < 0.001, data not shown).

**TABLE 3.**
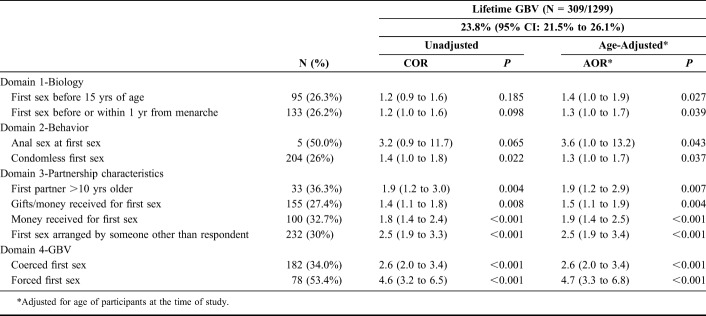
Association Between First Sex and Lifetime GBV Among Women Aged 14–24 Years in Mombasa, Kenya

**TABLE 4.**
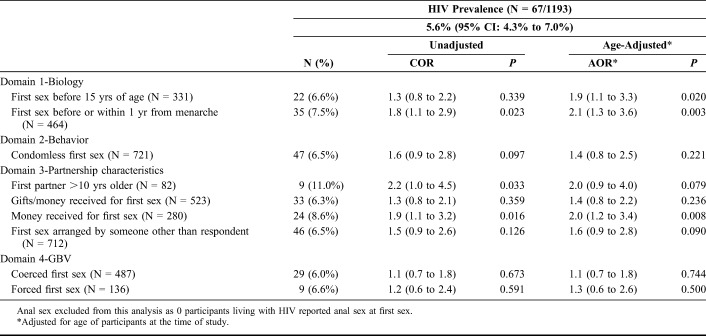
Association Between First Sex and HIV Prevalence Among Women Aged 14–24 Years in Mombasa, Kenya

The following first sex vulnerabilities were associated with GBV (Table 3): first sex before or within 1 year from menarche (COR 1.2, 95% CI: 1.0 to 1.6; AOR 1.4, 95% CI: 1.0 to 1.9, *P* = 0.027); a condomless first sex (COR 1.4, 95% CI: 1.0 to 1.8; AOR 1.3, 95% CI: 1.0 to 1.7, *P* = 0.039); an older first sex partner (COR 1.9, 95% CI: 1.2 to 3.0; AOR 1.9, 95% CI: 1.2 to 2.9, *P* = 0.007); the exchange of money or gifts at first sex (COR 1.4, 95% CI: 1.1 to 1.8; AOR 1.5, 95% CI: 1.1 to 1.9, *P* = 0.004); a first sex event arranged by someone other than themselves (COR 2.5, 95% CI: 1.9 to 3.3; AOR 2.5, 95% CI: 1.9 to 3.4, *P* < 0.001); and both coerced (COR 2.6, 95% CI: 2.0 to 3.4; AOR 2.6, 95% CI: 2.0 to 3.4, *P* < 0.001) and forced first sex events (OR 4.6, 95% CI: 3.2 to 6.5; AOR 4.7, 95% CI: 3.3 to 6.8, *P* < 0.001). Only after adjusting for age were the following also associated with GBV: age less than 15 years at first sex (AOR 1.4, 95% CI: 1.0 to 1.9, *P* = 0.027) and anal sex with first sex (AOR 3.6, 95% CI: 1.0 to 13.2, *P* = 0.037).

As shown in Table 4, participants living with HIV were 2-fold more likely to report first sex before or within 1 year of menarche (COR 1.8, 95% CI: 1.1 to 2.9; AOR 2.1, 95% CI: 1.3 to 3.6, *P* = 0.003) and receiving money for their first sex event (COR 1.9, 95% CI: 1.1 to 3.2, AOR 2.0, 95% CI: 1.2 to 3.4, *P* = 0.008). After adjusting for age, first sex before age 15 was associated with HIV (AOR 1.9, 95% CI: 1.1 to 3.3, *P* = 0.020), whereas HIV prevalence only tended toward an association with an older first sex partner (COR 2.2, 95% CI: 1.0 to 4.5; AOR 2.0, 95% CI: 0.9 to 4.0, *P* = 0.079).

## DISCUSSION

We identified a high prevalence of HIV-associated vulnerabilities experienced at the time of first sex among AGYW in Mombasa, Kenya. Almost 3 of every 10 AGYW reported a first sex that was early (<15 years of age) and before or within a year of menarche, an exchange of gifts or money at first sex, and a coerced first sex. In general, AGYW engaged in SW experienced a higher prevalence of most of the vulnerabilities examined in this study, but prevalence was high across the 3 groups, with notable similarities among those currently engaged in SW and TS. Several vulnerabilities at first sex were associated with lifetime experience of GBV and HIV prevalence. Compared with AGYW who are HIV-uninfected, AGYW living with HIV were more likely to report early first sex (<15 years) and before or within a year of menarche, and to receive money in exchange for sex at their first sex event.

We found that HIV-associated vulnerabilities begin very early in the sexual life course of AGYW who frequent SW hotspots. Although the first sex event may not lead to a transmission event in and of itself, the vulnerabilities we explored are associated with increased susceptibility to HIV acquisition.^5,12,17,39–41^ There was a higher prevalence of most first sex vulnerabilities than previously reported and likely reflects a higher-risk subset of AGYW in our study compared with the previous surveys. For example, early first sex (<15 years) was reported by 12% of women aged 15–24 years sampled in a 2014 Kenya household survey, compared with almost 28% of AGYW in our study.^42^ Few data are available on the prevalence of sex before or within a year of menarche, but the nearly 40% prevalence in our study is almost 3-fold higher than reported in the few studies that have timed first sex in relation to menarche in sub-Saharan Africa.^14^ Four of 10 AGYW in our study reported receipt of gifts or money at first sex, compared with 2%–14% of AGYW who reported a lifetime history of receiving gifts or money in exchange for sex, from a recent review.^27^ The 41.2% prevalence of a coerced first sex in our study was generally higher than that reported in 7 other community-based or household studies in sub-Saharan Africa.^43–47^ By contrast, the prevalence of a forced first sex in our study is similar to 13.2% prevalence among AGYW aged 15–24 years sampled in the 2008–2009 Demographic Health Survey (a nationally representative household survey).^23^ Thus, the population identified in our survey is generally much more vulnerable to very early HIV risks than the wider population AGYW in Kenya.

Furthermore, the prevalence of first sex vulnerabilities was high across AGYW irrespective of their engagement in SW, TS, or CS, suggesting considerable overlap in early HIV risk across subsets of AGYW in Kenya. Indeed, vulnerabilities in the partnership domain (especially exchange of gifts or money at first sex) were frequent and similar between AGYW who currently engage in SW and TS. Up to 40% of AGYW engaged in CS reported the exchange of gifts or money at first sex. Reasons may include similar economic forces that shape a first sex event, and engagement in SW and TS^48^ as well as overlapping sexual networks.^49^ In addition, our data are right-censored: the cross-sectional snapshot of current engagement in SW, TS, or CS means that some women currently engaged in TS or CS may enter formal SW and self-identify as a sex worker later. However, we cannot infer that first sex experiences lead AGYW to frequent SW hotspots or to engage in SW, TS, or CS. Nonetheless, SW hotspots could be practical sites for HIV programmatic outreach and engagement with AGYW who experienced a higher probability of HIV-associated first sex vulnerabilities, and in particular, compared with a broader population of AGYW identified in household surveys.^42^

Our findings also suggest that experiences at first sex are an early marker of, or may influence, the trajectory of HIV-associated vulnerabilities experienced thereafter. Ours is among the first studies to examine first sex vulnerabilities with ongoing or future experience of GBV.^4,43^ As in our study, one previous study in Tanzania showed women who experienced a forced first sex event were nearly 6 times more likely to experience repeated sexual or physical violence.^46^ Indeed, repeated acts of violence perpetrated by more than one partner are commonly reported^47,50^ suggesting that GBV-associated HIV transmission may be compounded over an AGYW's sexual life course.^22^

Across the first sex vulnerabilities associated with HIV prevalence, the first sex event itself may have led to HIV acquisition or influenced future HIV risk. For example, first sex before or within a year of menarche captures a combination of early first sex and sexual sociobehavioral changes associated with menarche and the onset of physical puberty.^14,51^ Menarche has been associated with school absenteeism and leaving school (especially when facilities are lacking to support menstrual hygiene),^14,52^ which in turn is associated with increased economic needs and TS, and subsequent sexually transmitted infections/HIV risk.^53–55^ An older first sex partner may become a longer-term intimate partner and, as shown in our study, is also associated with increased prevalence of GBV, which in turn is associated with increased HIV risk.^20^ The prevalence of first paid sex was similar among AGYW engaged in SW and TS. Yet, HIV prevalence was 3-fold higher among those in SW, suggesting that a paid first sex likely influences ongoing risk (which is highest among AGYW in SW).

In the context of HIV prevention, perhaps the most commonly examined first sex vulnerability in sub-Saharan Africa is the early first sex,^4^ with delay of sexual debut recommended as a population-level HIV prevention measure.^56–58^ In concert with findings from previous studies,^4,5,11,12^ early first sex (<15 years) was associated with both GBV and HIV in our study, but only after adjusting for age—suggesting that this first sex vulnerability and its influence on future GBV or HIV risk varies by birth cohorts, and key next steps will include examining first sex vulnerabilities by birth cohorts.

Our findings further support the inclusion of structural interventions including sexual health education, violence reduction, and economic empowerment to women before they become sexually active, and indeed, before achieving menarche. Interventions designed to influence prepubescent and early sexual life course include school-based programs^56^ and community-wide structural programs focused on addressing gender norms to delay sexual debut and reduce GBV.^59–62^ More recently, there is a growing focus on integrating sexual and reproductive behavioral and structural interventions (eg, conditional and unconditional cash transfer) within youth friendly health services.^63,64^ The findings could also be used to help identify AGYW who may be most at risk of ongoing vulnerabilities. In Kenya's Fast Track Plan to End HIV and AIDS Among Adolescents and Young People,^3^ a core component includes screening AGYW for sexual and reproductive health. Inclusion of first sex screening questions across the 4 domains examined here, with particular focus on vulnerabilities associated with the 2 outcomes we studied (lifetime GBV and HIV prevalence), could help to optimize sexual risk screening and identify those who may be at highest risk of prevalent HIV infection and future HIV risk.

Study limitations include the use of recall data on first sex events, which are subject to measurement bias. Data from repeated-measures studies in sub-Saharan Africa suggest that 30%–56% of self-reported estimates of age at first sex may be unreliable, although the measurement errors did not influence estimates of the median age of first sex.^65^ The data are also subject to social desirability bias resulting from the personal nature of questions around first sex and our use of face-to-face interviews—leading to potential under-reporting of vulnerabilities. Similarly, our classification of subgroups (SW, TS, and CS) relied on self-reported and disclosed partnership types, which could lead to misclassification of participants. We performed a cross-sectional study and thus cannot infer causation between events at first sex and lifetime GBV or HIV infection. Future work includes additional multivariate analyses to identify overlap in first sex vulnerabilities and potential mediators of HIV prevalence to disentangle the pathways potentially influenced by experiences at first sex, and qualitative studies to better explore the context surrounding experiences leading up to and risk factors for first sex vulnerabilities.^66^

## CONCLUSIONS

AGYW in Kenya, particularly those who frequent SW hotspots, experience high rates of HIV-associated vulnerabilities at first sex. Although we cannot infer causality, our findings suggest that first sex vulnerabilities are either a marker of—or potentially mediate—an AGYW's long-term trajectory of HIV-associated vulnerabilities and HIV risk. HIV prevention programs could use information about first sex to better understand the types and timing of vulnerabilities faced by AGYW, and how these vulnerabilities influence HIV acquisition over one's sexual life course. In doing so, programs can and should adapt and revise their strategies to provide targeted and timely interventions to decrease very early HIV risks.
